# MiR-125 Family in Cardiovascular and Cerebrovascular Diseases

**DOI:** 10.3389/fcell.2021.799049

**Published:** 2021-12-02

**Authors:** Yang Wang, Jing Tan, Lu Wang, Gaiqin Pei, Hongxin Cheng, Qing Zhang, Shiqi Wang, Chengqi He, Chenying Fu, Quan Wei

**Affiliations:** ^1^ Department of Rehabilitation Medicine Center, West China Hospital, Sichuan University, Chengdu, China; ^2^ Key Laboratory of Rehabilitation Medicine in Sichuan Province, Chengdu, China; ^3^ Department of Ultrasound Medicine, Binzhou People’s Hospital, Binzhou, China; ^4^ National Clinical Research Center for Geriatrics, West China Hospital, Sichuan University, Chengdu, China; ^5^ Aging and Geriatric Mechanism Laboratory, West China Hospital, Sichuan University, Chengdu, China

**Keywords:** cardiovascular and cerebrovascular diseases, mir-125, atherosclerosis, myocardial ischemia, ischemia-reperfusion, ischemic stroke, mesenchymal stem cell

## Abstract

Cardiovascular and cerebrovascular diseases are a serious threaten to the health of modern people. Understanding the mechanism of occurrence and development of cardiovascular and cerebrovascular diseases, as well as reasonable prevention and treatment of them, is a huge challenge that we are currently facing. The miR-125 family consists of hsa-miR-125a, hsa-miR-125b-1 and hsa-miR-125b-2. It is a kind of miRNA family that is highly conserved among different species. A large amount of literature shows that the lack of miR-125 can cause abnormal development of the cardiovascular system in the embryonic period. At the same time, the miR-125 family participates in the occurrence and development of a variety of cardiovascular and cerebrovascular diseases, including myocardial ischemia, atherosclerosis, ischemia-reperfusion injury, ischemic stroke, and heart failure directly or indirectly. In this article, we summarized the role of the miR-125 family in the development and maturation of cardiovascular system, the occurrence and development of cardiovascular and cerebrovascular diseases, and its important value in the current fiery stem cell therapy. In addition, we presented this in the form of table and diagrams. We also discussed the difficulties and challenges faced by the miR-125 family in clinical applications.

## Introduction

Cardiovascular disease is one of the leading causes of death in the world. A retrospective study from 204 countries around the world found that from 1990 to 2019, the total number of cardiovascular diseases increased from 271 million to 523 million, and the number of cardiovascular deaths increased from 12.1 million to 18.6 million (Roth et al., 2020). Therefore, we should actively explore the occurrence and development mechanism of cardiovascular disease, which is of great significance to alleviate the pressure of health system and improve the quality of life on a global scale.

Although it has been nearly 30 years since the first microRNA (miRNA) was discovered, the research on miRNA is still hot. Earlier studies found that miRNA expression was tissue-specific, that is, specific miRNAs were expressed only in specific cells or tissues (Lagos-Quintana et al., 2002). It was discovered that most miRNAs could be expressed in different organs or cells, and some of them were highly expressed among different species (Ludwig et al., 2016). Many studies have shown that miRNAs play an important regulatory role in the occurrence and development of diseases, and sometimes even in different stages in the same disease. Many miRNAs were highly conserved between different species (Perge et al., 2017). In different species, this provides us with the possibility to study whether the same miRNA plays the same role in the same disease, including cardiovascular and cerebrovascular diseases. For example, in both early and late stages, miR-19a/19b could protect cardiomyocytes in mice after myocardial infarction (MI) (Gao et al., 2019). Therapeutic silencing miR-146b-5p improved cardiac remodeling in porcine and mouse model of MI (Liao et al., 2021). MiR-21 from exosomes in human cardiomyocytes could be used to treat MI injury models in mice (Qiao et al., 2019).

**FIGURE 1:**
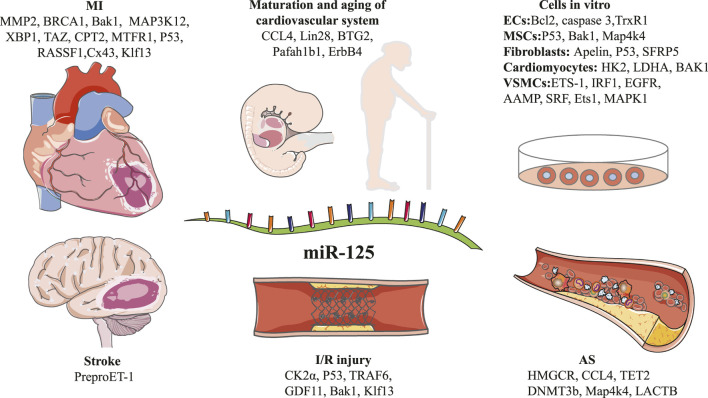
Schematic diagram of the target genes of miR-125 family in different types of disease pathogenesis.

**FIGURE 2:**
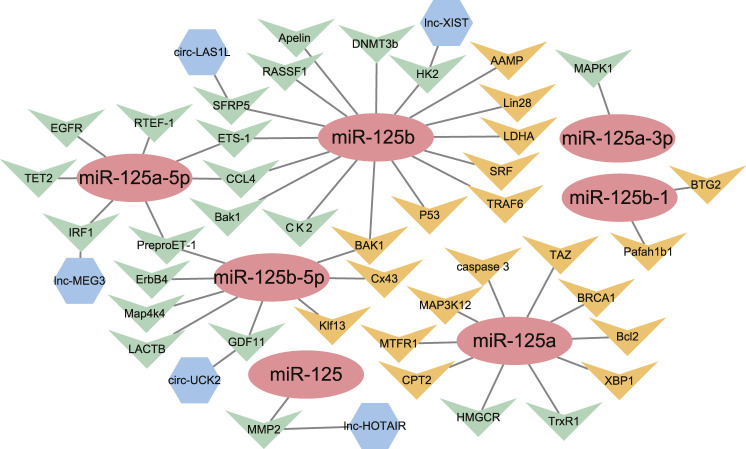
The network of miR-125 family members with their upstream genes and downstream genes. (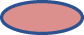) = miRNA-125 family members; (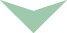) = Downstream target genes verified by luciferase assay; (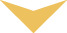) = Downstream target genes without luciferase assay; (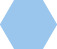) = Upstream target genes verified by luciferase assay.

MiR-125 family is widely expressed in mammals and its sequence is highly conserved. It is composed of three homologs hsa-miR-125a, hsa-miR-125b-1 and hsa-miR-125b-2 (Sun et al., 2013). hsa-miR-125a was discovered to be located at 19q13, while hsa-miR-125b-1 was verified on chromosomes 11q23 and hsa-miR-125b-2 on chromosomes 21q21 (Rodriguez et al., 2004). The miR-125 family plays an important role in the growth and development of animals, as well as the occurrence and development of cancer (Kim et al., 2016; Wang et al., 2019a). In addition, the role of miR-125 in cardiovascular and cerebrovascular diseases cannot be ignored. This article will review the role and mechanism of miR-125 family in the occurrence and development of cardiovascular and cerebrovascular diseases, including the role of the miR-125 family in the development and maturation of cardiovascular system, the occurrence and development of cardiovascular and cerebrovascular diseases, and its value in the stem cell therapy. Furthermore, we presented this in the form of tables and diagrams (Table1; Figures 1, 2). Finally, we also discussed the challenges faced by the miR-125 family in clinical applications in the future.

**TABLE 1:** Summary of studies investigating the regulators and effectors of miR‐125 family in cardiovascular and cerebrovascular diseases.

Reference	miRNA	Target cells/tissues/organs	Disease or phenotype	Intervention	Experimental setting	Species	Target genes
Li et al. (2018)	miR-125	H9c2	Oxidative stress	H_2_O_2_	*In vitro*	Rat	MMP2
Gródecka-Szwajkiewicz et al. (2020)	miR-125	Plasma	Premature birth	N	*In vivo*	Human	N
Díaz et al. (2017)	miR-125a	Myocardial cells	I/R injury	I/R, Urocortin	*In vitro*, *In vivo*	Rat	BRCA1, MAP3K12, XBP1, TAZ, CPT2, MTFR1
Svensson et al. (2014)	miR-125a	HUVECs, Arterial endothelial cells	Endothelial cell proliferation and viability	Growth factors	*In vitro*	Human	Bcl2, caspase-3
Maitrias et al. (2015)	miR-125a	Carotid plaque	Carotid plaque	N	*In vivo*	Human	N
Zhang and Niu, (2020)	miR-125a	Plasma	Acute ischemic stroke	N	*In vivo*	Human	N
Chen et al. (2018)	miR-125a	HUVECs	Oxidative stress	H_2_O_2_	*In vitro*	Human	TrxR1
Ye et al. (2020)	miR-125a	VSMCs	AS, VSMCs proliferation and migration	High glucose	*In vitro*	Rat	HMGCR
Li et al. (2020)	miR-125a	Plasma	Acute ischemic stroke	N	*In vivo*	Human	N
Hu et al. (2019)	miR-125a-3p	VSMCs	VSMCs proliferation and migration	Carotid artery balloon injury, Platelet derived growth factor	*In vitro*, *In vivo*	Rat	MAPK1
Wang et al. (2019b)	miR-125a-5p	VSMCs	AS	Oxidized low-density lipoprotein	*In vitro*	Human	CCL4
Zhou et al. (2021)	miR-125a-5p	VSMCs	VSMCs proliferation, migration and invasion	High glucose	*In vitro*	Rat	EGFR
Hendgen-Cotta et al. (2017)	miR-125a-5p	Heart	I/R injury	I/R,Nitrite	*In vivo*	Mouse	N
Che et al. (2014)	miR-125a-5p	Arterial endothelial cell	Aging	N	*In vitro*	Mouse	RTEF-1
Galluzzo et al. (2021)	miR-125a-5p	Serum	Advanced heart failure	N	*In vivo*	Human	N
Gareri et al. (2017)	miR-125a-5p	VSMCs, A10	Carotid artery balloon injury	Carotid artery balloon injury	*In vitro*, *In vivo*	Rat	ETS-1
Zheng et al. (2019)	miR-125a-5p	VSMCs	VSMCs proliferation	Platelet derived growth factor-BB, Vein graft	*In vitro*, *In vivo*	Rat	IRF1
Zhaolin et al. (2019)	miR-125a-5p	HUVECs	AS	Oxidized low-density lipoprotein	*In vitro*	Human	TET2
Kijpaisalratana et al. (2020)	miR-125a-5p, miR-125b-5p	Serum	Posterior circulation stroke/Peripheral vertigo	N	*In vivo*	Human	N
Tiedt et al. (2017)	miR-125a-5p, miR-125b-5p	Plasma	Acute ischemic stroke	N	*In vivo*	Human	N
Li et al. (2010)	miR-125a-5p, miR-125b-5p	H5V, b.END.3, VSMCs, NIH3T3	Oxidative stress	Oxidized low-density lipoprotein	*In vitro*, *In vivo*	Stroke-prone spontaneously hypertensive rats	PreproET-1
Ke et al. (2019)	miR-125a-5p, miR-125b-5p	Hippocampal tissues	I/R injury	I/R	Bioinformatics analysis	Rat	N
Wang et al. (2021)	miR-125b	VSMCs	Vascular smooth muscle cells proliferation	N	*In vitro*	Rat	AAMP, SRF
Nagpal et al. (2016)	miR-125b	Cardiac fibroblasts	Myocardial fibrosis	Angiotensin II, TGF-β2	*In vitro*, *In vivo*	Human, Mouse	Apelin, P53
Xu and Fang, (2021)	miR-125b	Myocardial cells	Diabetic cardiomyopathy/Myocardial cell death	High glucose	*In vitro*, *In vivo*	Human, Rat	HK2, LDHA
Zhang et al. (2021)	miR-125b	Myocardial cells	Heart failure/Cardiomyocyte apoptosis	Transverse aortic constriction	*In vitro*, *In vivo*	Mouse	Bak1
Liang et al. (2018)	miR-125b	PC12	I/R injury	I/R,OGD	*In vitro*	Rat	CK2α
Cheng et al. (2015)	miR-125b	Immune cells	Aging	N	*In vivo*	Human	CCL4
Sun et al. (2020)	miR-125b	Cardiac fibroblasts	AMI	Circ-LAS1L overexpression vector	*In vitro*	Human	SFRP5
Bie et al. (2016)	miR-125b	Cardiac fibroblasts	Cardiac fibroblasts growth and activation	N	*In vitro*	Human	SFRP5
Wang et al. (2014)	miR-125b	H9c2	I/R injury	I/R	*In vitro*, *In vivo*	Transgenic mice with overexpression of miR-125b + Rat	P53, Bak1, TRAF6
Wen et al. (2014)	miR-125b	VSMCs	VSMCs transdifferentiation and calcification	β-glycerophosphoric acid	*In vitro*	Rat	Ets1
Cao et al. (2016)	miR-125b	VSMCs	AS, VSMCs proliferation	Homocysteine, Methionine diet	*In vitro*, *In vivo*	Human, ApoE^−/-^ mouse	DNMT3b
Xiao et al. (2018)	miR-125b	MSCs, cardiomyocytes	MI, Autophagic Flux	MI, OGD, Co-culture	*In vitro*, *In vivo*	Mouse	P53
Wong et al. (2012)	miR-125b	ESCs	Embryo differentiation	N	*In vitro*	Human	Lin28
Fan et al. (2020)	miR-125b	H9c2, Cardiomyocytes	Cardiomyocyte injury	Hypoxia	*In vitro*	Rat	HK2
Ding et al. (2015)	miR-125b	Plasma	Coronary heart disease	N	*In vivo*	Human	N
Zhu et al. (2018)	miR-125b	Bone marrow mesenchymal stem cells, H9C2	MI	MI, Hypoxia, Co-culture, Reactive dibenzylcyclootyne	*In vitro*, *In vivo*	Mouse	P53, Bak1
Xiaochuan et al. (2020)	miR-125b	Myocardial cells	AMI	AMI, Adenoviruses containing RASSF1 siRNA, hypoxia	*In vivo*	Rat	RASSF1
Chen et al. (2021)	miR-125b-1	Heart	Birth defects	Cardiac-specific miR-125b-1 KO	*In vivo*	Cardiac specific miR-125b-1 KO mouse	BTG2, Pafah1b1
Szabó et al. (2020)	miR-125b-1-3p	Heart	Hypercholesterolemia, I/R injury	Special Diet, I/R	*In vitro*, *In vivo*	Rat	N
Deng et al. (2015)	miR-125b-2	Embryonic stem cells, E14TG2A	Birth defects	All-trans-retinoic acid	*In vitro*	Mouse	N
Dufeys et al. (2021)	miR-125b-5p	Cardiac fibroblasts	MI/Myocardial fibrosis	Myofibroblasts -specific AMPKα1 KO	*In vitro*, *In vivo*	Human, AMPKα1 KO mouse	Cx43
Nazari-Shafti et al. (2020)	miR-125b-5p	MSCs extracellular vehicles	N	N	*In vitro*	Human	N
Ben‐Zvi et al. (2020)	miR-125b-5p	Serum	Systolic heart failure	N	*In vivo*	Human	N
Lee et al. (2015)	miR-125b-5p	Embryonic stem cells, H9	Embryonic stem cells maturation	Co-culture	*In vitro*	Human, Mouse, Rat	ErbB4
Chen et al. (2020)	miR-125b-5p	HT-22	I/R injury	Oxygen Glucose Deprivation	*In vitro*	Mouse	GDF11
Bayoumi et al. (2018)	miR-125b-5p	HL-1, H9c2, Ventricular cardiomyocytes	AMI, I/R injury	AMI, I/R, Carvedilol	*In vitro*, *In vivo*	Mouse, Rat	Bak1, Klf13
Jia et al. (2016)	miR-125b-5p	Plasma	Acute myocardial infarction	N	*In vivo*	Human	N
Lin et al. (2021)	miR-125b-5p	Bone marrow mesenchymal stem cells, VSMCs, Aortic tissues	AS	High fat diet, Tail vein injection	*In vitro*, *In vivo*	Apoe^−/-^ mouse	Map4k4
Lu et al. (2016)	miR-125b-5p	THP-1, Atherosclerotic plaques	AS	LPS	*In vitro*, *In vivo*	Human	LACTB

## MiR-125 Family and the Maturation and Aging of Cardiovascular System

It was reported that when miR-125b-1 was specifically knocked out in the mouse heart, the mortality rate of perinatal mice was as high as 60%. Even in the surviving mice, their hearts were hypertrophy to varying degrees, and the mitochondria of the cardiomyocytes of these mice experienced varying degrees of morphological changes and loss of function in terms of morphology and function (Chen et al., 2021). Coincidentally, overexpression of miR-125b-2 in mouse embryonic stem cells (ESCs) inhibited the differentiation of mouse ESCs into endoderm and ectoderm, but it did not affect mesoderm differentiation, self-renewal and proliferation of mouse ESCs (Deng et al., 2015). Since the mesoderm was the origin of heart development, this study also indirectly showed that miR-125b-2 may play a role in maintaining the normal development of the heart in early mouse embryos. If murine and human embryonic-stem-cell-derived cardiomyocytes (m/hESC-CMs) were co-cultured with endothelial cells or endothelial cell lysates, it could improve the maturity and increase the expression of cardiomyocyte maturation markers in m/hESC-CMs. The reason for this phenomenon was that four miRNAs targeting ErbB4, including miR-125b-5p, overexpressed in m/hESC-CMs during the period when they were co-cultured with endothelial cells (Lee et al., 2015). This was probably because miRNAs from endothelial cells were released in the form of cell vesicles and absorbed by m/hESC-CMs during the co-cultivation process, thus increasing the maturity of m/hESC-CMs.

In another study on human ESCs (Wong et al., 2012), researchers found that miR-125b was an important regulator of human ESCs differentiation and development (including myocardium). Overexpression of miR-125b led to early heart disease. The upregulation of transcription factors, GATA4 and Nkx2-5, accelerated the progress of human embryonic stem cell-derived myocardial precursors to the phenotype of embryonic cardiomyocytes. In recent years, with the popularization of second-generation sequencing technology, more and more studies have shown that when the body was in a damaged state, large numbers of miRNAs would be released into the circulatory system from the damaged part (Cheng et al., 2019). Therefore, when the myocardium was damaged, the miRNA in the blood could be used as a marker of heart damage to a certain extent (Akat et al., 2014). Dorota Gródecka-Szwajkiewicz et al. analyzed the miRNA profile of umbilical cord blood of the eripheral blood mononuclear cells during the delivery of term infants and preterm infants. Many angiogenesis related miRNAs, including miR-125, decreased significantly in cord blood miRNAs of preterm infants. This may increase the risk of abnormal development and function of the cardiovascular system after these premature infants reached adulthood (Gródecka-Szwajkiewicz et al., 2020).

With the continuous aging, various components in the cell will change, and the cell function will also degrade. The increased expression of miR-125a in arterial endothelial cells of aging mice could regulate angiogenesis by targeting RTEF-1 and regulating the expression of eNOS and VEGF. The low expression of miR-125a in endothelial cells may be the “youth code” that maintained the normal operation of endothelial cells (Che et al., 2014). In addition, compared with the immune cells of young people, miR-125b was lowly expressed in the elderly. The expression of CCL4 was negatively correlated with the expression of miR-125b. CCL4 was an important chemokine of immune cells, which may be one of the reasons why aging had lower immunity compared with young people (Cheng et al., 2015).

## MiR-125 Family and Ischemia-Reperfusion Injury

After analyzing GSE82146 in the GEO database, Hong Ke et al. found that miR-125a and miR-125b may be the key genes that mediate ischemia-reperfusion (I/R) damage (Ke et al., 2019). In another study on I/R injury, more than half of the changes in miRNA expression including miR-125a-3p occurred in the nitrite treatment group compared with the control group 30 min after myocardial ischemia and 5 min after reperfusion (Hendgen-Cotta et al., 2017). These studies showed the high sensitivity of miR-125 family to I/R injury, which also provided a favorable reference value for miR-125 family to evaluate the therapeutic effect of I/R injury in the future. NF-κB signal pathway is the key to mediate myocardial injury after I/R injury. Overexpression of miR-125b in I/R injured mice could effectively reduce I/R-induced cardiomyocyte apoptosis, caspase-3/7 and caspase-8 activities, and prevent the activation of NF-κB pathway after I/R injury (Wang et al., 2014). In the rat I/R injury model, miR-125b could inhibit the expression of CK2α and regulate the CK2α/NADPH oxidation signal pathway to protect the rat brain from I/R damage directly (Liang et al., 2018).

In addition to directly targeting downstream mRNA for regulation, ceRNA bonded by miRNA and circRNA influence the expression of mRNA to produce regulation. Cheng Luo et al. found that the expression of circPVT1 in rats with I/R injury increased significantly. It could inhibit the expression of miR-125b and miR-200a by targeting them, increase cardiomyocytes apoptosis after I/R injury, and weaken their protective effect on heart muscles (Luo et al., 2021). MiR-125a, together with miR-139 and miR-324, could cooperate with urocortin to protect rat myocardium after I/R injury (Díaz et al., 2017). By targeting MAPK1, miR-125a-3p could also inhibited intimal thickening and the function of vascular smooth muscle cells (VSMCs), thereby reducing the degree of restenosis (Hu et al., 2019). In addition, miR-125a could participate in the proliferation of endothelial cells by acting on Bcl2, caspase-3 and TrxR1 *in vitro* experiments (Svensson et al., 2014; Chen et al., 2018). Interventional therapy is the traditional treatment for vascular stenosis. However, I/R injury and restenosis after interventional therapy have always been a major problem for clinicians. All the above studies provide potential targets for the treatment of I/R injury and vascular restenosis after injury from the gene level.

## MiR-125 Family and Myocardial Ischemia

Currently, the number of studies of miR-125b is the most among miRNAs related to myocardial ischemia in the miR-125 family. Researchers found that the expression of miR-125b in the plasma of coronary heart disease patients was lower than that of non-coronary heart disease patients. As the Gensini score increased, the level of miR-125b reduced significantly (Ding et al., 2015). Diabetic cardiomyopathies is a special type of heart disease. miR-125b and miR-34a could protect cardiomyocytes in high glucose environment. Their action on the HK2 in glucose metabolism and LDHA in lactate metabolism respectively inhibited the glucose metabolism, glucose uptake and lactate metabolism of cardiomyocytes (Xu and Fang, 2021).

Hypercholesterolemia is one of the causes of myocardial ischemia. Ischemic preconditioning could up-regulate the expression of miR-125b-1-3p and activate cardiac self-protection mechanisms. However, rats with hypercholesterolemia could attenuate the up-regulation of miR-125b-1-3p level through ischemic preconditioning, which was related to the loss of cardioprotection (Szabó et al., 2020). Myocardial infarction (MI) is one of the main types of myocardial ischemia. Accurate and timely diagnosis of acute myocardial infarction (AMI) is particularly important for a good prognosis of patients. After analyzing the plasma miRNA data of AMI patients, it was found that miR-125b-5p and miR-30d-5p could be used to diagnose AMI effectively. Compared with the existing diagnostic methods of CK-MB, cTnI and myoglobin, the diagnostic performance of miRNA could be comparable to or even exceed the potential of existing diagnostic indicators (Jia et al., 2016). All of these contribute a new idea to the diagnosis of myocardial ischemia in our clinical work. Cardiomyocyte apoptosis is a common pathological process after myocardial ischemia. Up-regulation of miR-125b in cardiomyocytes could reduce the protein levels of apoptosis-related markers c-caspase-3 and Bax significantly, and increase the expression of anti-apoptotic protein Bcl-2 to increase the survival rate of cardiomyocytes (Zhang et al., 2021). In addition, miR-125b could also inhibit cardiomyocytes apoptosis by inhibiting the expression of RASSF1 and KLF3 (Bayoumi et al., 2018; Xiaochuan et al., 2020). lncRNA is another non-coding RNA which has the similar function with circRNA. It can form ceRNA with miRNA to weaken the post-transcriptional modification effect of miRNA on mRNA. In H9C2 cells, lncRNA-XIST could form ceRNA with miR-125b to affect the downstream HEK2 gene, which could weaken the cardioprotective effect of miR-125b and result in cardiomyocytes damage (Fan et al., 2020).

Myocardial fibrosis is an important cause of decreased heart function after myocardial ischemia. When heart was injured, the Ang II-TGF-β axis could influence the expression of miR-125b, and then inhibit the expression of apelin and p53, leading to the proliferation of fibroblasts and the conversion of fibroblasts to myofibroblasts. The final outcome of the increase in miR-125b level was the occurrence of myocardial remodeling (Nagpal et al., 2016). The effect of miR-125a did seem to be opposite to that of miR-125b. Similarly, in H9C2 cells, miR-125a mimics reduced the expression of MMP2 and the proliferation of it, while promoting its apoptosis. However, lnc-HOTAIR could restore the expression of MMP2 by targeting miR-125a. At the same time, it could also promote the proliferation and survival of H9C2 (Li et al., 2018). In cardiac fibroblasts from patients with AMI, circ-LAS1L could bind to miR-125b to relieve the inhibitory effect on downstream SFRP5. Furthermore, it could inhibit the activation, proliferation and migration, and promote apoptosis of cardiac fibroblasts (Bie et al., 2016; Sun et al., 2020). Compared with WT mice, the MI model of AMPKα1 knocked out conditionally showed more severe myocardial remodeling. Interfering with AMPKα1 expression in fibroblasts could also reduce the expression of Cx43 protein significantly. However, deletion of AMPKα1 could only reduce the activity of Cx43 promoter by about 40%, which was inconsistent with the expression of Cx43 protein. By using a preliminary quantitative PCR microRNA array, the authors showed that AMPKα1, in addition to binding directly to Cx43, could perform post-transcriptional control of Cx43 by upregulating miR-125b in its own absence (Dufeys et al., 2021).

## MiR-125 Family and Atherosclerosis

As we all know, atherosclerosis (AS) is a chronic and complex disease involving multiple factors, and it is one of the main causes of coronary heart disease, myocardial ischemia, and cerebral infarction. By comparing the miRNA in symptomatic with asymptomatic atherosclerotic plaques after surgical resection Pierre Maitrias et al. found that miR-125a was significantly different between the two groups (Maitrias et al., 2015). This implies that miR-125a plays an important role in the changes of atherosclerotic plaques at different stages of the disease. Macrophages are important participants in the AS process. It is of great significance to reduce the aggregation of macrophages and protect damaged endothelial cells from inflammation in the control of AS. MCP-1 is an important chemokine for macrophages. In THP-1 macrophage cell line, miR-125b-5p could inhibit the expression of MCP-1 by targeting LACTB and attenuate the chemotaxis of macrophages (Lu et al., 2016). Pyroptosis is a new mode of programmed cell death that has been discovered and confirmed in recent years, and its development is often accompanied the release of massive inflammatory factors. TET2 was an important member of the TET enzyme family and played an important role in epigenetics (Shen et al., 2018). After treating the endothelial cell surface with oxLDL *in vitro*, the increased expression of miR-125a-5p could reduce the expression of TET2. Inactivation of TET2 could result in abnormal DNA methylation, NF-κB nuclear transposition, inflammatory response, and subsequent pyroptosis (Zhaolin et al., 2019).

With the development of the disease, VSMCs subjected to inflammatory stimulation will proliferate, migrate, and invade. This is one of the causes of plaque formation, arterial calcification, and stenosis. As we mentioned before, miR-125b had a targeting relationship with CCL4 (Cheng et al., 2015). Interestingly, in VSMCs, miR-125a also had a targeting relationship with CCL4. This combination could inhibit the expression of NLRP3 and alleviate the inflammatory process (Wang et al., 2019b). Ping Wen et al. treated primary rat VSMCs cultured *in vitro* with β-glycerophosphate and found that the β-glycerophosphate promoted the phenotypic transition and calcification of VSMCs. Besides, they found that the expression of miR-125b decreased significantly. After the VSMCs transfected with miR-125b mimics, they could resist β-glycerophosphate-mediated cell differentiation and calcification (Wen et al., 2014). The effect on inhibiting the proliferation of VSMCs of miR-125b was also confirmed in previous study. In the cell model of homocysteine-induced VSMCs proliferation, miR-125b could counteract the proliferation of VSMCs by targeting DNMT3b and mediating p53 DNA methylation (Cao et al., 2016). In addition, miR-125b could also inhibit the proliferation and migration of VSMCs by inhibiting the expression levels of AAMP and SRF (Wang et al., 2021). The function of miR-125a on VSMCs was the same as miR-125b, and both of them could inhibit the proliferation and migration of VSMCs. miR-125a could play a role by inhibiting EST1, which was related to cell proliferation and migration in the PDGF-BB pathway (Gareri et al., 2017). In addition, miR-125a could also work by targeting IRF1, EGFR and HMGcr in VSMCs (Zheng et al., 2019; Ye et al., 2020; Zhou et al., 2021).

## MiR-125 Family and Other Cardiovascular and Cerebrovascular Diseases

At present, many of the cardiovascular and cerebrovascular patients would develop heart failure gradually as the diseases progress (Sulo et al., 2020). In patients with heart failure, the expression of miR-125b would rise significantly (Ben-Zvi et al., 2020). And in patients with advanced heart failure, there were significant differences in the expression of three miRNAs including miR-125a-5p, which were related to the composite end point of cardiac death, cardiac transplantation, or mechanical circulatory support implantation (Galluzzo et al., 2021). These studies had important predictive reference value for the prognosis of patients with heart failure.

In terms of pathogenesis, there are many similarities between ischemic stroke and myocardial ischemia. For example, in addition to being a marker of myocardial ischemia, the miR-125 family could also be used as a potential biomarker for acute vertigo, posterior circulation stroke and acute ischemic stroke (Tiedt et al., 2017; Kijpaisalratana et al., 2020). In a large case-control study (210 participants in the control group, 210 participants in the acute ischemic stroke group), the researchers found that the expression of miR-125a decreased in the plasma of the experimental group. The reason for this decline was that lnc-NEAT1 inhibited its expression (Li et al., 2020). The other lnc-ITSN1-2 inhibited the expression of miRNA such as miR-125a via inactivating its anti-angiogenesis and anti-inflammatory effects. The ultimate result was changes in alter vascular structure as well as inflammation related NF-κB pathway and TRL pathway activation (Zhang and Niu, 2020). In addition, the reduction of miR-125a-5p and miR-125b-5p expression was also related to the expression of preproET-1 in the aorta of stroke-susceptible spontaneous hypertensive rats (SHR-SPs) (Li et al., 2010). This provided new and strong evidence for the miR-125 family to participate in the maintenance of vascular homeostasis in the body. In the process of ischemic stroke, circ-UCK2 could act as an endogenous miR-125b-5p sponge to inhibit the activity of miR-125b-5p, which in turn led to an increase in GDF11 expression and improved neuronal damage subsequently (Chen et al., 2020).

## MiR-125 and Mesenchymal Stem Cells and Their Extracellular Vesicles

The current treatments for cardiovascular and cerebrovascular diseases, such as interventions and drugs, have certain therapeutic effects. However, in many cases, conventional treatments cannot save the dying tissues. Mesenchymal stem cells (MSCs) are a kind of stem cells with multiple differentiation potentials and a promising treatment for cardiovascular and cerebrovascular diseases. By far, stem cell therapy has been partially applied to the clinical work of cardiovascular and cerebrovascular diseases (Hu et al., 2011). miR-125b-5p was highly expressed in the miRNA profiles of extracellular vesicles isolated from MSCs derived from cord blood and adipose tissues (Nazari-Shafti et al., 2020). This is the basis of MSCs and their vesicles in the treatment of cardiovascular and cerebrovascular diseases.

In the ApoE−/− mice model of AS, the researchers found that the exosomal miR-125b-5p from mouse bone marrow mesenchymal stem cells (BMSCs) inhibited the formation of atherosclerotic plaques by inhibiting the expression of Map4k4 (Lin et al., 2021). P53 was an important apoptosis-regulating gene in organisms, and it could regulate the apoptosis process of cells in a variety of ways (Hafner et al., 2019). Studies have found that transplantation of MSCs or their exosomes could effectively inhibit the autophagy flux, cell death and P53 gene expression of cardiomyocytes after MI. And the therapeutic effect of the MSCs-exosome treatment group is significantly better than that of the MSCs-exosome-antimiR-125b group. Therefore, stem cells and their exosomes were likely to inhibit autophagy flux and target P53 through miR-125b, thereby inhibiting the apoptosis process of cardiomyocytes mediated by P53 gene and protecting cardiomyocytes (Xiao et al., 2018). It has been reported that when they were used to treat ischemic mouse body models, MSCs could improve their ability to promote functional angiogenesis after undergoing a hypoxia process (Huang et al., 2013). So, if the MSCs are pretreated with hypoxia, will their ability to treat cardiovascular and cerebrovascular diseases be improved? The answer is yes. MI mice treated with exosomes of BMMCs after 72 h of hypoxia culture had a significant reduction in the area of MI. Next-generation sequencing showed that hypoxia treatment could significantly increase the content of miR-125b-5p in exosomes of BMMCs, and this mechanism of action was due to the ability of miR-125b to inhibit the expression of pro-apoptotic genes P53 and BAK1 in cardiomyocytes (Zhu et al., 2018). This study proved the highly effective treatment effect of miR-125 family members on MI again.

## Conclusion

More and more studies have shown that miR-125 family is related to the development and differentiation of mammalian embryonic heart. Furthermore, they were found played an important role in diseases and pathophysiological processes such as, coronary heart disease, MI, I/R injury, stroke, myocardial fibrosis, endothelial cell injury and myocardial cell apoptosis. However, in different diseases and different pathological processes, the same miR-125 family members play different roles. That is very interesting. For example, overexpression of miR-125b in cardiomyocytes can inhibit cardiomyocyte apoptosis and inflammatory response in pathological state to protect cardiomyocytes. But at the same time, miR-125b is also a regulator of cardiac fibrosis. Its overexpression in cardiac fibroblasts can enhance their proliferation and reduce their apoptosis. Therefore, excessive miR-125b will aggravate myocardial fibrosis and myocardial remodeling under pathological conditions, destroy the original morphological structure of the heart, increase the difficulty of neovascularization, and aggravate the apoptosis of cardiomyocytes in the damaged area.

As mentioned above, if miR-125 is used as a treatment target of cardiovascular and cerebrovascular diseases in clinical practice, further studies have to take the following two points into account. Firstly, because some miR-125 family members have ‘two sides’ in the role of cardiovascular and cerebrovascular diseases, we should find the best ‘balance point’ between harmful and beneficial to give full play to its maximum treatment and circumvent its negative effects. Secondly, in the future, it may not be enough to only study the optimal therapeutic dose of miR-125 in the process of diagnosis and treatment. It is also necessarily need to find a suitable miR-125 vector that can target our target cells for “precision treatment”. Only in this way can we give full play to the optimal therapeutic effect of miR-125 family members.

## References

[B1] Akat K. M. Moore-McGriff D. Morozov P. Brown M. Gogakos T. Correa Da Rosa J. (2014). Comparative RNA-Sequencing Analysis of Myocardial and Circulating Small RNAs in Human Heart Failure and Their Utility as Biomarkers. Proc. Natl. Acad. Sci. 111 (30), 11151–11156. 10.1073/pnas.1401724111 25012294 PMC4121804

[B2] Bayoumi A. S. Park K.-m. Wang Y. Teoh J.-p. Aonuma T. Tang Y. (2018). A Carvedilol-Responsive microRNA, miR-125b-5p Protects the Heart from Acute Myocardial Infarction by Repressing Pro-apoptotic Bak1 and Klf13 in Cardiomyocytes. J. Mol. Cell. Cardiol. 114, 72–82. 10.1016/j.yjmcc.2017.11.003 29122578 PMC5800989

[B3] Ben‐Zvi I. Volinsky N. Grosman‐Rimon L. Haviv I. Rozen G. Andria N. (2020). Cardiac‐peripheral Transvenous Gradients of microRNA Expression in Systolic Heart Failure Patients. ESC Heart Fail. 7 (3), 835–843. 10.1002/ehf2.12597 32253819 PMC7261589

[B4] Bie Z.-d. Sun L.-y. Geng C.-l. Meng Q.-g. Lin X.-j. Wang Y.-f. (2016). MiR-125b Regulates SFRP5 Expression to Promote Growth and Activation of Cardiac Fibroblasts. Cell Biol Int 40 (11), 1224–1234. 10.1002/cbin.10677 27592695

[B5] Cao C. Zhang H. Zhao L. Zhou L. Zhang M. Xu H. (2016). miR-125b Targets DNMT3b and Mediates P53 DNA Methylation Involving in the Vascular Smooth Muscle Cells Proliferation Induced by Homocysteine. Exp. Cel Res. 347 (1), 95–104. 10.1016/j.yexcr.2016.07.007 27426728

[B6] Che P. Liu J. Shan Z. Wu R. Yao C. Cui J. (2014). miR‐125a‐5p Impairs Endothelial Cell Angiogenesis in Aging Mice via RTEF ‐1 Downregulation. Aging cell 13 (5), 926–934. 10.1111/acel.12252 25059272 PMC4331751

[B7] Chen C.-Y. Lee D. S. Choong O. K. Chang S.-K. Hsu T. Nicholson M. W. (2021). Cardiac-specific microRNA-125b Deficiency Induces Perinatal Death and Cardiac Hypertrophy. Sci. Rep. 11 (1), 2377. 10.1038/s41598-021-81700-y 33504864 PMC7840921

[B8] Chen F. Liu H. Wu J. Zhao Y. (2018). miR-125a Suppresses TrxR1 Expression and Is Involved in H2O2-Induced Oxidative Stress in Endothelial Cells. J. Immunol. Res. 2018, 1–7. 10.1155/2018/6140320 PMC6129346 30225271

[B9] Chen W. Wang H. Feng J. Chen L. (2020). Overexpression of circRNA circUCK2 Attenuates Cell Apoptosis in Cerebral Ischemia-Reperfusion Injury via miR-125b-5p/GDF11 Signaling. Mol. Ther. - Nucleic Acids 22, 673–683. 10.1016/j.omtn.2020.09.032 33230465 PMC7585838

[B10] Cheng M. Yang J. Zhao X. Zhang E. Zeng Q. Yu Y. (2019). Circulating Myocardial microRNAs from Infarcted Hearts Are Carried in Exosomes and Mobilise Bone Marrow Progenitor Cells. Nat. Commun. 10 (1), 959. 10.1038/s41467-019-08895-7 30814518 PMC6393447

[B11] Cheng N. L. Chen X. Kim J. Shi A. H. Nguyen C. Wersto R. (2015). MicroRNA‐125b Modulates Inflammatory Chemokine CCL4 Expression in Immune Cells and its Reduction Causes CCL4 Increase with Age. Aging cell 14 (2), 200–208. 10.1111/acel.12294 25620312 PMC4364832

[B12] Deng S. Zhang Y. Xu C. Ma D. (2015). MicroRNA-125b-2 Overexpression Represses Ectodermal Differentiation of Mouse Embryonic Stem Cells. Int. J. Mol. Med. 36 (2), 355–362. 10.3892/ijmm.2015.2238 26059631 PMC4501654

[B13] Díaz I. Calderón-Sánchez E. Toro R. D. Ávila-Médina J. de Rojas-de Pedro E. S. Domínguez-Rodríguez A. (2017). miR-125a, miR-139 and miR-324 Contribute to Urocortin protection against Myocardial Ischemia-Reperfusion Injury. Sci. Rep. 7 (1), 8898. 10.1038/s41598-017-09198-x 28827743 PMC5566224

[B14] Ding X.-Q. Ge P.-C. Liu Z. Jia H. Chen X. An F.-H. (2015). Interaction between microRNA Expression and Classical Risk Factors in the Risk of Coronary Heart Disease. Sci. Rep. 5, 14925. 10.1038/srep14925 26446730 PMC4597355

[B15] Dufeys C. Daskalopoulos E.-P. Castanares-Zapatero D. Conway S. J. Ginion A. Bouzin C. (2021). AMPKα1 Deletion in Myofibroblasts Exacerbates post-myocardial Infarction Fibrosis by a Connexin 43 Mechanism. Basic Res. Cardiol. 116 (1), 10. 10.1007/s00395-021-00846-y 33564961 PMC7873123

[B16] Fan J.-L. Zhu T.-T. Xue Z.-Y. Ren W.-Q. Guo J.-Q. Zhao H.-Y. (2020). lncRNA-XIST Protects the Hypoxia-Induced Cardiomyocyte Injury through Regulating the miR-125b-Hexokianse 2 axis. *In Vitro* Cell.Dev.Biol.-Animal 56 (4), 349–357. 10.1007/s11626-020-00459-0 32415544

[B17] Galluzzo A. Gallo S. Pardini B. Birolo G. Fariselli P. Boretto P. (2021). Identification of Novel Circulating microRNAs in Advanced Heart Failure by Next‐generation Sequencing. ESC Heart Fail. 8 (4), 2907–2919. 10.1002/ehf2.13371 33934544 PMC8318428

[B18] Gao F. Kataoka M. Liu N. Liang T. Huang Z.-P. Gu F. (2019). Therapeutic Role of miR-19a/19b in Cardiac Regeneration and protection from Myocardial Infarction. Nat. Commun. 10 (1), 1802. 10.1038/s41467-019-09530-1 30996254 PMC6470165

[B19] Gareri C. Iaconetti C. Sorrentino S. Covello C. De Rosa S. Indolfi C. (2017). miR-125a-5p Modulates Phenotypic Switch of Vascular Smooth Muscle Cells by Targeting ETS-1. J. Mol. Biol. 429 (12), 1817–1828. 10.1016/j.jmb.2017.05.008 28502794

[B20] Gródecka-Szwajkiewicz D. Ulańczyk Z. Zagrodnik E. Łuczkowska K. Rogińska D. Kawa M. P. (2020). Differential Secretion of Angiopoietic Factors and Expression of MicroRNA in Umbilical Cord Blood from Healthy Appropriate-For-Gestational-Age Preterm and Term Newborns-In Search of Biomarkers of Angiogenesis-Related Processes in Preterm Birth. Ijms 21 (4), 1305. 10.3390/ijms21041305 PMC7072966 32075190

[B21] Hafner A. Bulyk M. L. Jambhekar A. Lahav G. (2019). The Multiple Mechanisms that Regulate P53 Activity and Cell Fate. Nat. Rev. Mol. Cel Biol 20 (4), 199–210. 10.1038/s41580-019-0110-x 30824861

[B22] Hendgen-Cotta U. B. Messiha D. Esfeld S. Deenen R. Rassaf T. Totzeck M. (2017). Inorganic Nitrite Modulates miRNA Signatures in Acute Myocardial *In Vivo* Ischemia/reperfusion. Free Radic. Res. 51 (1), 91–102. 10.1080/10715762.2017.1282158 28090786

[B23] Hu S. Liu S. Zheng Z. Yuan X. Li L. Lu M. (2011). Isolated Coronary Artery Bypass Graft Combined with Bone Marrow Mononuclear Cells Delivered through a Graft Vessel for Patients with Previous Myocardial Infarction and Chronic Heart Failure. J. Am. Coll. Cardiol. 57 (24), 2409–2415. 10.1016/j.jacc.2011.01.037 21658561

[B24] Hu W. Chang G. Zhang M. Li Y. Yin L. Huang Y. (2019). MicroRNA-125a-3p Affects Smooth Muscle Cell Function in Vascular Stenosis. J. Mol. Cell. Cardiol. 136, 85–94. 10.1016/j.yjmcc.2019.08.014 31499051

[B25] Huang C.-C. Chen D.-Y. Wei H.-J. Lin K.-J. Wu C.-T. Lee T.-Y. (2013). Hypoxia-induced Therapeutic Neovascularization in a Mouse Model of an Ischemic Limb Using Cell Aggregates Composed of HUVECs and cbMSCs. Biomaterials 34 (37), 9441–9450. 10.1016/j.biomaterials.2013.09.010 24054844

[B26] Jia K. Shi P. Han X. Chen T. Tang H. Wang J. (2016). Diagnostic Value of miR-30d-5p and miR-125b-5p in Acute Myocardial Infarction. Mol. Med. Rep. 14 (1), 184–194. 10.3892/mmr.2016.5246 27176713 PMC4918561

[B27] Ke H. Zhang X. Cheng L. Fan Y. Xiao S. Ma Y. (2019). Bioinformatic Analysis to Explore Key Genes Associated with Brain Ischemia-Reperfusion Injury in Rats. Int. J. Neurosci. 129 (10), 945–954. 10.1080/00207454.2019.1595615 30889366

[B28] Kijpaisalratana N. Nimsamer P. Khamwut A. Payungporn S. Pisitkun T. Chutinet A. (2020). Serum miRNA125a-5p, miR-125b-5p, and miR-433-5p as Biomarkers to Differentiate between Posterior Circulation Stroke and Peripheral Vertigo. BMC Neurol. 20 (1), 372. 10.1186/s12883-020-01946-3 33038923 PMC7547489

[B29] Kim K.-H. Seo Y.-M. Kim E.-Y. Lee S.-Y. Kwon J. Ko J.-J. (2016). The miR-125 Family Is an Important Regulator of the Expression and Maintenance of Maternal Effect Genes during Preimplantational Embryo Development. Open Biol. 6 (11), 160181. 10.1098/rsob.160181 27906131 PMC5133438

[B30] Lagos-Quintana M. Rauhut R. Yalcin A. Meyer J. Lendeckel W. Tuschl T. (2002). Identification of Tissue-specific MicroRNAs from Mouse. Curr. Biol. 12, 735–739. 10.1016/s0960-9822(02)00809-6 12007417

[B31] Lee D. S. Chen J.-H. Lundy D. J. Liu C.-H. Hwang S.-M. Pabon L. (2015). Defined MicroRNAs Induce Aspects of Maturation in Mouse and Human Embryonic-Stem-Cell-Derived Cardiomyocytes. Cel Rep. 12 (12), 1960–1967. 10.1016/j.celrep.2015.08.042 26365191

[B32] Li D. Yang P. Xiong Q. Song X. Yang X. Liu L. (2010). MicroRNA-125a/b-5p Inhibits Endothelin-1 Expression in Vascular Endothelial Cells. J. Hypertens. 28 (8), 1646–1654. 10.1097/HJH.0b013e32833a4922 20531225

[B33] Li L. Zhang M. Chen W. Wang R. Ye Z. Wang Y. (2018). LncRNA-HOTAIR Inhibition Aggravates Oxidative Stress-Induced H9c2 Cells Injury through Suppression of MMP2 by miR-125. Acta Biochim. Biophys. Sinica 50 (10), 996–1006. 10.1093/abbs/gmy102 30239560

[B34] Li P. Duan S. Fu A. (2020). Long Noncoding RNA NEAT1 Correlates with Higher Disease Risk, Worse Disease Condition, Decreased miR‐124 and miR‐125a and Predicts Poor Recurrence‐free Survival of Acute Ischemic Stroke. J. Clin. Lab. Anal. 34 (2), e23056. 10.1002/jcla.23056 31721299 PMC7031604

[B35] Liang Y. Xu J. Wang Y. Tang J.-Y. Yang S.-L. Xiang H.-G. (2018). Inhibition of MiRNA-125b Decreases Cerebral Ischemia/Reperfusion Injury by Targeting CK2α/NADPH Oxidase Signaling. Cell Physiol Biochem 45 (5), 1818–1826. 10.1159/000487873 29510389

[B36] Liao Y. Li H. Cao H. Dong Y. Gao L. Liu Z. (2021). Therapeutic Silencing miR-146b-5p Improves Cardiac Remodeling in a Porcine Model of Myocardial Infarction by Modulating the Wound Reparative Phenotype. Protein Cell 12 (3), 194–212. 10.1007/s13238-020-00750-6 32845445 PMC7895884

[B37] Lin F. Zhang S. Liu X. Wu M. (2021). Mouse Bone Marrow Derived Mesenchymal Stem Cells-Secreted Exosomal microRNA-125b-5p Suppresses Atherosclerotic Plaque Formation via Inhibiting Map4k4. Life Sci. 274, 119249. 10.1016/j.lfs.2021.119249 33652034

[B38] Lu J.-B. Yao X.-X. Xiu J.-C. Hu Y.-W. (2016). MicroRNA-125b-5p Attenuates Lipopolysaccharide-Induced Monocyte Chemoattractant Protein-1 Production by Targeting Inhibiting LACTB in THP-1 Macrophages. Arch. Biochem. Biophys. 590, 64–71. 10.1016/j.abb.2015.11.007 26603571

[B39] Ludwig N. Leidinger P. Becker K. Backes C. Fehlmann T. Pallasch C. (2016). Distribution of miRNA Expression across Human Tissues. Nucleic Acids Res. 44 (8), 3865–3877. 10.1093/nar/gkw116 26921406 PMC4856985

[B40] Luo C. Ling G.-x. Lei B.-f. Feng X. Xie X.-y. Fang C. (2021). Circular RNA PVT1 Silencing Prevents Ischemia-Reperfusion Injury in Rat by Targeting microRNA-125b and microRNA-200a. J. Mol. Cell. Cardiol. 159, 80–90. 10.1016/j.yjmcc.2021.05.019 34097926

[B41] Maitrias P. Metzinger-Le Meuth V. Massy Z. A. M'Baya-Moutoula E. Reix T. Caus T. (2015). MicroRNA Deregulation in Symptomatic Carotid Plaque. J. Vasc. Surg. 62 (5), 1245–1250. 10.1016/j.jvs.2015.06.136 26238333

[B42] Nagpal V. Rai R. Place A. T. Murphy S. B. Verma S. K. Ghosh A. K. (2016). MiR-125b Is Critical for Fibroblast-To-Myofibroblast Transition and Cardiac Fibrosis. Circulation 133 (3), 291–301. 10.1161/CIRCULATIONAHA.115.018174 26585673 PMC5446084

[B43] Nazari-Shafti T. Z. Neuber S. Duran A. G. Exarchos V. Beez C. M. Meyborg H. (2020). MiRNA Profiles of Extracellular Vesicles Secreted by Mesenchymal Stromal Cells-Can They Predict Potential Off-Target Effects. Biomolecules 10 (9), 1353. 10.3390/biom10091353 PMC7565205 32971982

[B44] Perge P. Nagy Z. Decmann Á. Igaz I. Igaz P. (2017). Potential Relevance of microRNAs in Inter-species Epigenetic Communication, and Implications for Disease Pathogenesis. RNA Biol. 14 (4), 391–401. 10.1080/15476286.2016.1251001 27791594 PMC5411124

[B45] Qiao L. Hu S. Liu S. Zhang H. Ma H. Huang K. (2019). microRNA-21-5p Dysregulation in Exosomes Derived from Heart Failure Patients Impairs Regenerative Potential. J. Clin. Invest. 129 (6), 2237–2250. 10.1172/jci123135 31033484 PMC6546482

[B46] Rodriguez A. Ashurst J. L. Bradley A. (2004). Identification of Mammalian microRNA Host Genes and Transcription Units. Genome Res. 14, 1902–1910. 10.1101/gr.2722704 15364901 PMC524413

[B47] Roth G. A. Mensah G. A. Johnson C. O. Addolorato G. Ammirati E. Baddour L. M. (2020). Global Burden of Cardiovascular Diseases and Risk Factors, 1990-2019: Update from the GBD 2019 Study. J. Am. Coll. Cardiol. 76 (25), 2982–3021. 10.1016/j.jacc.2020.11.010 33309175 PMC7755038

[B48] Shen Q. Zhang Q. Shi Y. Shi Q. Jiang Y. Gu Y. (2018). Tet2 Promotes Pathogen Infection-Induced Myelopoiesis through mRNA Oxidation. Nature 554 (7690), 123–127. 10.1038/nature25434 29364877

[B49] Sulo G. Sulo E. Jørgensen T. Linnenberg A. Prescott E. Tell G. S. (2020). Ischemic Heart Failure as a Complication of Incident Acute Myocardial Infarction: Timing and Time Trends: A National Analysis Including 78,814 Danish Patients during 2000-2009. Scand. J. Public Health 48 (3), 294–302. 10.1177/1403494819829333 30813840

[B50] Sun L. y. Zhao J. c. Ge X. m. Zhang H. Wang C. m. Bie Z. d. (2020). Circ_LAS1L Regulates Cardiac Fibroblast Activation, Growth, and Migration through miR‐125b/SFRP5 Pathway. Cell Biochem Funct 38 (4), 443–450. 10.1002/cbf.3486 31950540

[B51] Sun Y.-M. Lin K.-Y. Chen Y.-Q. (2013). Diverse Functions of miR-125 Family in Different Cell Contexts. J. Hematol. Oncol. 6, 6. 10.1186/1756-8722-6-6 23321005 PMC3566921

[B52] Svensson D. Gidlöf O. Turczyńska K. M. Erlinge D. Albinsson S. Nilsson B.-O. (2014). Inhibition of microRNA-125a Promotes Human Endothelial Cell Proliferation and Viability through an Antiapoptotic Mechanism. J. Vasc. Res. 51 (3), 239–245. 10.1159/000365551 25116893

[B53] Szabó M. R. Gáspár R. Pipicz M. Zsindely N. Diószegi P. Sárközy M. (2020). Hypercholesterolemia Interferes with Induction of miR-125b-1-3p in Preconditioned Hearts. Ijms 21 (11), 3744. 10.3390/ijms21113744 PMC7312064 32466450

[B54] Tiedt S. Prestel M. Malik R. Schieferdecker N. Duering M. Kautzky V. (2017). RNA-seq Identifies Circulating miR-125a-5p, miR-125b-5p, and miR-143-3p as Potential Biomarkers for Acute Ischemic Stroke. Circ. Res. 121 (8), 970–980. 10.1161/CIRCRESAHA.117.311572 28724745

[B55] Wang J. K. Wang Z. Li G. (2019). MicroRNA-125 in Immunity and Cancer. Cancer Lett. 454, 134–145. 10.1016/j.canlet.2019.04.015 30981762

[B56] Wang J. Wu Q. Yu J. Cao X. Xu Z. (2019). miR-125a-5p I-nhibits the E-xpression of NLRP3 by T-argeting CCL4 in H-uman V-ascular S-mooth M-uscle C-ells T-reated with ox-LDL. Exp. Ther. Med. 18 (3), 1645–1652. 10.3892/etm.2019.7717 31410121 PMC6676174

[B57] Wang X. Chen S. Gao Y. Yu C. Nie Z. Lu R. (2021). MicroRNA-125b I-nhibits the P-roliferation of V-ascular S-mooth M-uscle C-ells I-nduced by P-latelet-derived G-rowth F-actor BB. Exp. Ther. Med. 22 (2), 791. 10.3892/etm.2021.10223 34093747 PMC8170660

[B58] Wang X. Ha T. Zou J. Ren D. Liu L. Zhang X. (2014). MicroRNA-125b Protects against Myocardial Ischaemia/reperfusion Injury via Targeting P53-Mediated Apoptotic Signalling and TRAF6. Cardiovasc. Res. 102 (3), 385–395. 10.1093/cvr/cvu044 24576954 PMC4030511

[B59] Wen P. Cao H. Fang L. Ye H. Zhou Y. Jiang L. (2014). miR-125b/Ets1 axis Regulates Transdifferentiation and Calcification of Vascular Smooth Muscle Cells in a High-Phosphate Environment. Exp. Cel Res. 322 (2), 302–312. 10.1016/j.yexcr.2014.01.025 24486760

[B60] Wong S. S. Y. Ritner C. Ramachandran S. Aurigui J. Pitt C. Chandra P. (2012). miR-125b Promotes Early Germ Layer Specification through Lin28/let-7d and Preferential Differentiation of Mesoderm in Human Embryonic Stem Cells. PloS one 7 (4), e36121. 10.1371/journal.pone.0036121 22545159 PMC3335794

[B61] Xiao C. Wang K. Xu Y. Hu H. Zhang N. Wang Y. (2018). Transplanted Mesenchymal Stem Cells Reduce Autophagic Flux in Infarcted Hearts via the Exosomal Transfer of miR-125b. Circ. Res. 123 (5), 564–578. 10.1161/CIRCRESAHA.118.312758 29921652

[B62] Xiaochuan B. Qianfeng J. Min X. Xiao L. (2020). RASSF1 Promotes Cardiomyocyte Apoptosis after Acute Myocardial Infarction and Is Regulated by miR‐125b. J. Cel Biochem 121 (1), 489–496. 10.1002/jcb.29236 31595551

[B63] Xu C.-r. Fang Q.-j. (2021). A Inibição Do Metabolismo da Glicose por miR-34a e miR-125b Protege contra a Morte Celular de Cardiomiócitos Causada por Hiperglicemia. Arquivos brasileiros de cardiologia 116 (3), 415–422. 10.36660/abc.20190529 33909769 PMC8159564

[B64] Ye D. Lou G. H. Li A. C. Dong F. Q. Chen G. P. Xu W. W. (2020). MicroRNA-125a-mediated R-egulation of the M-evalonate S-ignaling P-athway C-ontributes to H-igh G-lucose-induced P-roliferation and M-igration of V-ascular S-mooth M-uscle C-ells. Mol. Med. Rep. 22 (1), 165–174. 10.3892/mmr.2020.11077 32319638 PMC7248521

[B65] Zhang B. Mao S. Liu X. Li S. Zhou H. Gu Y. (2021). MiR-125b Inhibits Cardiomyocyte Apoptosis by Targeting BAK1 in Heart Failure. Mol. Med. 27 (1), 72. 10.1186/s10020-021-00328-w 34238204 PMC8268255

[B66] Zhang Y. Niu C. (2020). The Correlation of Long Non‐coding RNA Intersectin 1‐2 with Disease Risk, Disease Severity, Inflammation, and Prognosis of Acute Ischemic Stroke. J. Clin. Lab. Anal. 34 (2), e23053. 10.1002/jcla.23053 31647141 PMC7031635

[B67] Zhaolin Z. Jiaojiao C. Peng W. Yami L. Tingting Z. Jun T. (2019). OxLDL Induces Vascular Endothelial Cell Pyroptosis through miR‐125a‐5p/TET2 Pathway. J. Cel Physiol 234 (5), 7475–7491. 10.1002/jcp.27509 30370524

[B68] Zheng X. Wu Z. Xu K. Qiu Y. Su X. Zhang Z. (2019). Interfering Histone Deacetylase 4 Inhibits the Proliferation of Vascular Smooth Muscle Cells via Regulating MEG3/miR-125a-5p/IRF1. Cell Adhes. Migration 13 (1), 41–49. 10.1080/19336918.2018.1506653 PMC6527374 30156956

[B69] Zhou H. Lin S. Hu Y. Guo D. Wang Y. Li X. (2021). miR-125a-5p and miR-7 I-nhibits the P-roliferation, M-igration and I-nvasion of V-ascular S-mooth M-uscle C-ell by T-argeting EGFR. Mol. Med. Rep. 24 (4). 10.3892/mmr.2021.12347 PMC8383035 34396443

[B70] Zhu L.-P. Tian T. Wang J.-Y. He J.-N. Chen T. Pan M. (2018). Hypoxia-elicited Mesenchymal Stem Cell-Derived Exosomes Facilitates Cardiac Repair through miR-125b-Mediated Prevention of Cell Death in Myocardial Infarction. Theranostics 8 (22), 6163–6177. 10.7150/thno.28021 30613290 PMC6299684

